# Exploring Concepts of Understanding in the Working Practice of Speech and Language Therapists in the UK: A Preliminary Survey

**DOI:** 10.1111/1460-6984.70302

**Published:** 2026-08-04

**Authors:** K. Bunning, S. Buell, L. Flannery, E. Manzi, N. Grove

**Affiliations:** ^1^ School of Health Sciences, Faculty of Medicine & Health University of East Anglia, Chancellor's Drive Norwich Norfolk UK; ^2^ School of Health Sciences University of Dundee, Nethergate Dundee UK; ^3^ Rix Inclusive Research, University of East London, Docklands Campus University of East London London, Greater London UK

**Keywords:** conceptualisation, speech and language therapy, survey, practice, theory, understanding

## Abstract

**Background:**

All communication involves the expression and the reception of information, both of which are considered by speech and language therapists (SLTs) when assessing and planning intervention for language difficulties. Comprehension, or understanding, has to be inferred, whereas expressive behaviours are directly observable. Possibly because of the level of inference, views and assessments of a person's understanding often vary: between different practitioners, professionals, family members and carers. The aim of the research was to examine how and why such different perspectives occur and how they are managed. There were three research questions: (1) How is understanding conceptualised and addressed from the perspectives of: (a) SLT practitioners and (b) SLT educators? (2) Are discrepant views on a person's understanding recognised and encountered in SLT practice? and (3) if so, how are such differences explained and navigated in practice?

**Methods:**

The research design used a survey methodology involving two bespoke online questionnaires aimed at: (i) SLT practitioners; and (ii) SLT educators. Purposive convenience samples were recruited via UK‐based pre‐registration programmes in Speech and Language Therapy and the Clinical Excellence Networks (CENs) of the Royal College of Speech and Language Therapists (RCSLT). The online questionnaires included demographic information followed by a mixture of closed and free‐field questions exploring conceptualisation and assessment of understanding. Descriptive statistics were applied to closed question responses, and summative content analysis to the free‐field responses.

**Results:**

A total of 80 completed questionnaires were returned: (i) SLT practitioners = 77; (ii) SLT educators = 3. Understanding was defined in different ways. Approaches to assessment focused on vocabulary and semantics, language processing, and responses to different communicative modalities. Differing views on a person's understanding were recognised to be a common issue encountered by SLTs, which were mainly associated with parents, partners and family members rather than fellow professionals.

**Conclusions:**

SLTs seem to vary in the ways they conceptualise and address the question of understanding in their practice, and the findings confirm anecdotal suggestions that differences of opinion with other professionals, family members and carers are relatively common. Receptive communication, therefore, emerges as a contested field, which has implications for how successfully and thoroughly SLTs are able to plan and deliver effective interventions. Further clarification is needed regarding the relevant information and guidance provided in pre‐registration training. Implementation science may provide some useful guidance as to the marrying of theory and research evidence.

**WHAT THIS PAPER ADDS:**

*What is already known on the subject*

- Language involves both expression and comprehension, the one process being observable whereas the other is inferred. Various models of understanding are proposed, which speech and language therapists can apply in their therapeutic practice, but we know little about which they choose and why. Furthermore, there is anecdotal report of disagreements occurring between assessments made by the speech and language therapist and those of other professionals, family members and caregivers.

*What this paper adds to the existing knowledge*

- The current study is the first reported survey into how understanding is conceptualised and operationalised in the practice of speech and language therapists in the UK. The findings revealed the different ways that understanding was defined with assessment approaches generally focusing on components within the architecture of language and communication. It appears that differing views on a person's understanding are often encountered by speech and language therapists.

*What are the potential or clinical implications of this work?*

- This survey suggests that more work needs to be done to establish a unified theory of understanding to underpin speech and language therapy practice: one that combines social cognitive processes and psycholinguistic processes.

## 1 Background

The problem of translating research findings into effective practice is a long‐recognised issue in the science of intervention (Douglas and Burshnic 2019; McCurtin and Clifford 2015; Thome et al. 2020). Studies have focused on the factors that interfere with the transition and influence the judgement of clinicians. In this paper, we are interested in a vital precursor, that of differences in how a problem or process is conceptualised and how these may impact on the practices of assessment and intervention.

Of the two dimensions of communication, understanding has always been recognised as more challenging to assess than observable expressive behaviours (Bloom 1974; Sperber and Wilson 2012). The domain of understanding encompasses all relevant aspects of language (e.g. vocabulary, grammar and linguistic pragmatics) represented by spoken, manual and visual symbols, as well as contextual pragmatics, which includes the ability to interpret embodied communication and environmental cues (Van Dijk 2008). Ferstl (2021) argues that ‘language serves communication, and communication … is situated in linguistic and social contexts’ (p. 37). Put plainly, communication does not happen in a void; the influences between setting and language use are bidirectional. One complication is that different views about an individual's level of understanding—potentially at variance with that of the speech and language therapist (SLT)—may be espoused by others, including fellow professionals (e.g. nursing staff, teachers) family members and carers, (Grove et al. 2000; Smith et al. 2020). Contrasting assessment findings may be attributable to different perspectives offered by functional communication in the setting (e.g. home, classroom, day centre) as compared to standardised testing which eliminates contextual cues. Given the interaction between language and context, it is not surprising that assessing a person's understanding is far from straightforward.

### 1.1 What Is Comprehension? Models and Frameworks

To start with, the terminology deployed by SLTs and other colleagues can be confusing. Terms such as *receptive language*, *comprehension* and *understanding* are used interchangeably. But are these terms synonymous? Or do the semantics differ in subtle ways? One website on bilingualism in childhood claims that ‘Receptive Language is the term used to describe everything a person understands’ (Bilingual Kidspot: https://bilingualkidspot.com/2022/12/09/what‐is‐receptive‐expressive‐language) a position which is emphatically rejected by Wallach and Ocampo (2021). These authors maintain that the term ‘receptive language’ fails to take account of the reality that comprehension is constructed in context, and suggest SLTs instead use ‘spoken/witten language comprehension’.

Models and processes are invoked in discussion of both developmental and acquired language difficulties. A traditional information processing model was expounded by Bishop (1997) whereby acoustic signals are translated into meaning, via word recognition, sentence recognition and inference from context and general knowledge. Bishop accepted that inference presents a challenge to a simple directional model (Sperber and Wilson 2012; Wilson and Bishop 2021). She was also critical of Fodor's modular theories (1983), at least in the context of child language acquisition. Griffin et al. (2020) considered comprehension as a multi‐faceted process informed by intrinsic and extrinsic factors. Intrinsic factors include sensory decoding; attention channelling and management; grammatical and lexical knowledge; working memory; and cognition interacting with past experiences, physical and psychosocial conditions (Wolvin 2009). Extrinsic factors are associated with the environment, which is defined in terms of ambient noise and visual levels, linguistic complexity, other speaker and stimuli characteristics. With regard to the comprehension of school‐aged children, Purse and Gardner (2013) concluded that effective communication assessment and profiling was informed by analytical assessment (speech perception, semantics, morphosyntax and pragmatics, working memory and metacognition) combined with functional assessment (the learning environment and associated processes).

With regard to the field of adult acquired aphasia, Wallace and colleagues (2022; p. 2404) define auditory comprehension briefly as ‘the ability to understand spoken language’ with the sequelae of aphasia being deficits in specific language components (phonology, semantics, syntax, and grammar), exacerbated by underlying cognitive damage to areas such as attention and working memory. The authors do not specify any particular model, but an information processing framework is implied by the way that language components are differentiated from more general cognitive mechanisms. Lwi and colleagues (2021) focus on spoken sentence and single word comprehension—what might be seen as the tip of the iceberg/fulcrum for SLTs. In relation to primary progressive aphasia, Dial et al. (2024) define auditory comprehension as the ‘perception and comprehension of auditorily presented stimuli’ (p. 273) within a hierarchical process of analysis that starts with acoustic input of the word, referred to as the spectrotemporal level, before mapping information onto abstract, speech‐specific sub‐lexical representations, which in turn are combined to form lexical‐phonological representations, which are then mapped to ‘lexical‐semantic representations’ (p. 273). The final level is where word features associated with meaning are activated at the semantic level (Hickok and Poeppel 2000).

Such hierarchies appear as linear; however, in discussions of speech perception, interactivity (or lack thereof) between and within levels of the hierarchy continues to be debated (e.g. McClelland et al. 2006; Samuel 2011; Sohoglu et al. 2012).

It can be seen, therefore, that the interactions between multiple aspects of comprehension have featured consistently in research and theory published in the last decades. As early as 1987, Crystal argued for such interactions to be taken into account in his ‘Bucket Theory’ of language, albeit with a focus on production. However, he also observed the tendency to associate absence of linguistic forms in production with a lack of competence (p.15). The Bucket Theory does not make the same predictions as the model by Terband et al. (2016), which suggests that if one language process is deficient, neighbouring processes are affected in a negative manner as well. In contrast, the Bucket Theory predicts that if a child with developmental language delay improves in one linguistic domain, their performance in other linguistic processes stagnates or decreases. So, this theory predicts trade‐offs between different linguistic processes, whereas the model by Terband and colleagues (2016) predicts positive relationships between different linguistic processes. However, the first two interactions proposed by Crystal (1987) could be explained by the model by Terband et al. (2016), because when the phonological decoding process receives more complex input (i.e. a complex grammatical structure), it is more likely to make errors, resulting in less fluent and more unintelligible utterances.

So far, the predominant way of conceptualising comprehension from the SLT or psychological perspective remains what Zwaan (2014) terms the latent, or default view, which adopts an abstract, symbolic view of cognition and a modular approach to language. However, the past 30 years have seen the concurrent development of other, more integrated, views of how understanding develops and operates: embodied comprehension (Dupont et al. 2023; Zwaan 2014); comprehension‐production synthesis (Pickering and Garrod 2013); multimodal gestalt recognition and multi‐level prediction (Holler and Levinson 2019) and the dialogical co‐construction of meaning (Linell 2014). One of the most significant contributions is Relevance Theory (Sperber and Wilson 2012), which suggests that two different processes, inference and decoding, are synthesised in the assignment of meaning. Relevance Theory is widely accepted and has been applied in various apposite contexts (Blakemore 2005; Buell 2017; Ferstl 2021; Grove 2005; Ifantidou and Wharton 2024; Leinonen and Kerbel 1999), but has rarely been cited by SLTs in the literature on comprehension.

In relation to modularity, there are outstanding questions regarding the roles of domain‐specific versus general executive processes in the act of comprehension (Holler and Levinson 2019; MacDonald 2022; Ryskin and Nieuwland 2023). With regard to context and social interpretation, Gee (2014) points out that all language is contextualised, so that the idea of assessing isolated language processes makes little sense. Similarly, Doedens and Metyard (2022) propose a real‐world functional approach that focuses on the multimodal, interactive and non‐linear nature of situated in‐person understanding. The picture is further complicated by the need to consider understanding of written texts and multimedia (Davoudi and Moghadam 2015; Parcalabescu et al. 2020), and of computational language (Elleström 2021; Khurana et al. 2023).

### 1.2 Understanding in Practice

In an exploration of SLT practice with preschool children, Morgan and Dipper (2018) found that the majority of the 245 SLTs who participated (89%) reported that comprehension was foundational and essential in their work. The authors note the complexity of how language functions, and the inadequacies of a simplified hierarchical model such as ‘the communication pyramid’, which depicts stages of language development founded on ‘attention and listening skills’. (p. 26). A review of school and speech and language therapy websites revealed the use and acceptance of the pyramid model, despite a lack of underpinning evidence (Morgan and Dipper 2018). They also found that therapists were rarely accessing research evidence or different frameworks and theories and were possibly relying instead on their intuitive experience, as also suggested by Boschuizen and Schmidt (2000). One reason for an over‐reliance on intuition might be the limited evidence on intervention effectiveness for children with spoken language difficulties (Law et al. 2004). A similar reliance on experience appears to underpin the report by Goldbart et al. (2014). They found that SLTs engaged in diverse practices with children and adults with profound and multiple intellectual disabilities, with limited recourse to evidence‐based practice. Respondents to their survey most frequently identified client‐centred factors as critical to their intervention planning decisions, rather than published research (Goldbart et al. 2014).

### 1.3 Intervention Approaches

Not only do models of comprehension differ markedly, but intervention programmes may also treat domains of language in somewhat different ways. For example, Wallace et al. (2022) found three distinct and different approaches in the field of aphasia to remediating deficits in auditory comprehension: using spoken modality only, using a multimodal approach, or indirect methods targeting associated non‐language processes such as attention and memory. Another approach is ‘mapping therapy’, which views comprehension difficulties as an inability to map between the meaning form and thematic roles, and the syntactic form of the sentence (Valinejad et al. 2024). Some interventions view comprehension from an integral perspective of communication partnerships and the interactions taking place. Founded on clear psychosocial principles, conversation analysis offers an approach to assessment and remediation of functional language in context (Horton et al. 2020). It has been used to address ‘trouble’ source and repair in everyday conversation sequences (Tetnowski et al. 2021; Wilkinson 1999). For example, Azios et al. (2023) reported improvements in mutual understanding between a person with aphasia and their marital partner through changes in gestural use and flexibility in conversation. Examples from the field of neuro‐developmental and intellectual disabilities field include the SCERTS model (Prizant et al. 2017) for autistic individuals and Storysharing (Grove 2014). Both approaches position comprehension within a social framework of collaborative meaning construction within the communication partnership. Similarly, ‘Video Interaction Guidance’ (www.videointeractionguidance.net) is also located in naturally occurring social interaction. It is most commonly used in adult‐child dyads to promote mutually attuned interactions (Rogers and Bond 2024).

Other interventions are founded on natural language development and used variously with pre‐school and school‐aged children with language delays and associated developmental conditions. They draw on a usage‐based theory of language acquisition centred around the utterance as a psycholinguistic unit (Tomasello 2000). For example, the Derbyshire Language Scheme (DLS) is a composite programme based on the level of linguistic demand of instructions. (Knowles and Masidlover 1982). Children progress through levels defined by the number of ‘Information Carrying Words (ICWs)’—from 1 to 4 or more—in a single sentence. Similar to the DLS, Building Early Sentences Therapy (BEST: McKean et al. 2013) manipulates the complexity of linguistic utterances at clause level to which the child is exposed (McKean et al. 2025). Marion Blank developed a classroom‐based approach to assessing understanding, pairing question levels (1–4, based on a theory of perceptual distance) with child responses: Appropriate, Inappropriate, Irrelevant, Incomplete or Nil response. Any response other than Appropriate signals there may be a problem (Blank et al. 1978).

### 1.4 Discrepant Perspectives

In the late 1990 and early 2000s, several research papers were published on the ways in which staff in residential homes and day services overestimated the understanding of persons with intellectual/learning disabilities (Banat et al. 2002; Bradshaw 2001; Kevan 2003; McConkey et al. 1999; Purcell et al. 2000). In contrast, Purse and Gardner (2013) found that teacher observation of children's functional comprehension in the classroom was commensurate with the outcomes of standardised speech and language therapy assessments. Miller et al. (2017) reported similar agreement between the standardised assessment and parental review of autistic children's comprehension. However, concerns have been raised about the accuracy of parental report (Ozonoff et al. 2011; Tomasello and Mervis 1994; Zapolski and Smith 2013). Although parents are often keen observers of their child's early development, limited knowledge of developmental milestones (Nordahl‐Hansen et al. 2014); a tendency to over‐focus on challenging or unusual behaviours (Zapolski and Smith 2013), and a reluctance to recognise their child's language delay (Ozonoff et al. 2011) may introduce bias to their report (Sachse and Von Suchodoletz 2008). In a comparison of parental and professional assessment of receptive language in pre‐school children with autistic spectrum disorder and other developmental delays, James et al. (2023) found parental assessments to be lower in the diagnostic groups with autistic spectrum disorders and autistic‐type features. Thus, the type of disorder may be a factor in assessment accuracy. Data from other client groups are more limited, so it is possible that differences of opinion amongst SLTs and professionals, family members and carers about someone's understanding are more prevalent with people who have neurodevelopmental and intellectual disabilities.

Since this period, very little research on understanding has appeared in the literature. This is a serious problem at a time where person‐centred care and collaborative decision‐making processes are prioritised in service provision (Forsgren et al. 2022). In their recent survey of communication problems of people with intellectual disabilities in Ireland, Smith and colleagues (2020) consider discrepancies between different stakeholder assessments of an individual's understanding to be a persistent issue. Why this matters is made clear when it comes to research. The Mental Capacity Act (DoH 2005) for England and Wales and the Adults with Incapacity (Scotland) Act (Scottish Parliament 2000), which underpin the involvement of individuals in life decisions, are founded on the presumption of capacity—as demonstrated by the ability to receive and understand information, to weigh up the implications of a decision and to communicate that decision—unless proven otherwise. It appears that different concepts of ‘understanding’ exist and may contribute to a lack of clarity for many families and professionals, including SLTs.

### 1.5 Research Aims

This study was conducted to examine how and why different interpretations of understanding arise across different professionals, family members and carers. A first step was to explore how the concept of understanding is represented within the professional practice and education of SLTs. A second step was to examine the occurrence of any discrepant perspectives, possible explanations and approaches to their management. The research was guided by the following questions:

1. How is understanding conceptualised and addressed from the perspectives of: (a) SLT practitioners and (b) SLT educators?
2. Are discrepant views on a person's understanding recognised and encountered within SLT practice.
3. If so, how are such differences explained and navigated in practice?

## 2 Methods

### 2.1 Design

The study adopted a survey methodology that involved bespoke online questionnaires generated in MS Forms and targeted at SLT practitioners and SLT educators. A follow‐up stage involving semi‐structured interviews with a sample of self‐nominated volunteers from the SLT practitioners group was also conducted. This study will be reported separately at a later date.

### 2.2 Ethics

The study received ethical approval from the Faculty of Medicine and Health Ethics Monitor at the University of East Anglia (ETH2324‐1590). Questionnaire responses were completed online and automatically anonymised. Data files were stored on the secure OneDrive in a folder with access only to team members.

### 2.3 Recruitment and Sample

A purposive‐convenience sample was targeted comprising: (1) SLT practitioners/members of Clinical Excellence Networks (CENs), who work with people with speech, language and communication difficulties, developmental and acquired across the lifespan; (2) SLT educators representing a range of relevant disciplines who are currently employed within SLT programmes at UK‐based universities.

Recruitment of SLT practitioners was conducted via the CENs registered to the Royal College of Speech and Language Therapists, UK. In particular, this involved those CENs focused on speech, language and communication (*N* = 85) and excluded CENs focused on dysphagia. A project flyer was emailed to the listed contact person for dissemination to their membership. The 85 CENs represented the clinical areas of aphasia; augmentative and alternative communication; autism; bilingualism; brain injury; children and young people; community and home care; deafness; dementia; developmental language disorder; learning disabilities; neurological; mental health; justice and legal; research; selective mutism; sensory impairment; and technology. Recruitment of SLT educators was conducted via each SLT programme at an institute of higher education in the UK (*N* = 22). A project flyer was emailed to the Course Director/Head of School/Department, who was asked to disseminate information to academic staff contributing to the taught programme for pre‐registration SLT students. Project information was displayed on the first few pages of the questionnaire, which was followed by a consent form. Once the participant recorded their consent, they were then invited to complete the questionnaire.

### 2.4 Data Collection

Two questionnaires were developed, one for each targeted group, that is, SLT practitioners and SLT educators. A team of academics and practitioners developed the questionnaire content drawing on their knowledge, expertise and practice in the fields of learning disabilities, aphasia and paediatrics. The questionnaire content was designed to be as neutral as possible by not aligning with any one published model. MS Forms software was used to construct the questionnaires. Demographic information was collected initially, followed by two sections containing 12 questions that were a mixture of closed questions with response options and free‐field questions on: Information on Understanding; Assessment of Understanding. Completion of the questionnaire was estimated to take approximately 20 min. The questionnaires were piloted with two academic SLT colleagues and one SLT practitioner.

### 2.5 Data Analysis

From the collected data in MS Forms, two outputs were generated automatically: (1) raw participant‐level data in an Excel spreadsheet; (2) a summary report with built‐in charts and descriptive statistics. Responses to closed questions were summated with raw figures and percentages reported. The exception was question 20, which asked the respondent to rate the frequency with which they assessed different aspects of comprehension using a four‐point semantic scale (usually‐often‐sometimes‐never). The number of respondents was reported for each aspect per point on the rating scale. Accompanying the raw data, a weighted score was reported by multiplying the raw figure (no. of respondents) by the numerical weighting of the individual point on the rating scale. This enabled report of the total weighted scores for each assessment aspect in rank order.

Summative content analysis (Hsieh and Shannon 2005) was applied to the free‐field responses. The free‐field responses were entered into a prepared Excel workbook with sheets focused on each of the key questions: different views about a person's understanding; assessment tools; and explaining understanding. The respondents’ free‐field responses were recorded in the lefthand column of an Excel spreadsheet. The first author, K.B. (background in developmental and learning disabilities) carried out the initial analysis. Each response was read closely and a first generation of themes was established based on the response content. The theme titles were inserted into the top cell of each column to the right of the column containing the respondents’ responses. Reference to a theme was indicated by inserting a ‘1’ into the relevant cell. The number of respondents for each theme was automatically summated in the bottom cell. Once the textual content had been reviewed several times against the listed themes, the themes were grouped homogeneously. A second review and analysis was then undertaken by the fourth author, E.M. (background in acquired language disorders). This was followed by a meeting to discuss any disagreements in the analyses until consensus was achieved. A final review involved checking for distinctiveness of each group composed of themes, making adjustments as required and summating the number of respondents at a grouped thematic level.

## 3 Results

The results are presented as follows: participant demographics, including years of experience, place of work, the nature of the work, client populations; conceptualisation of ‘understanding’, models that inform practice, and tools and frameworks used in assessment and intervention; and occurrence of discrepant views on understanding and how they are negotiated with other professionals, family members and carers. Summary tables are presented for the SLT practitioner data only (*N* = 77). SLT educator data (*N* = 3) are presented in supporting textual summaries.

### 3.1 Sample

SLT practitioners: There were 77 respondents, which included 5 managers; 70 SLT practitioners; 1 newly qualified SLT; and 1 ‘other, for example, recently retired’. The range of working years as an SLT was: 1–3 years (*n* = 8; 10 %); 4–9 years (*n* = 25; 32%); 10–15 years (*n* = 12; 17%); 16–29 years (*n* = 23; 30%); and 30+ years (*n* = 9; 12%). The place of work varied with some working in more than one setting: community (*n* = 35; 44.9%); hospital (*n* = 17; 21.8%); school/college (*n* = 14; 17.9); forensic unit (*n* = 11; 14.1%); pre‐school (*n* = 6; 7.7%); social services (*n* = 4; 5.1%); voluntary sector (*n* = 1; 1.3%). ‘Other’ settings were identified by 13 (16.6%) respondents, which included work within the youth justice system, specialist rehabilitation centre, neuro‐developmental team, gender identity out‐patient clinic and early years settings. Independent practice was declared by 16 (20.5%) respondents.

As shown in Table 1, there was a good representation of different clinical areas/specialisms across respondents. The majority of respondents worked in more than one area. Six respondents indicated ‘other’ for clinical group which included: mental health; selective mutism; generalised movement and health needs. Respondents tended to work across more than one client group and age range, distributed as follows: infants 0–2 years (*n* = 14; 18%); pre‐school 3–5 years (*n* = 23; 30%); children 11–18 years (*n* = 37; 48%); adults 18–64 years (*n* = 51; 66 %); adults 65+years (*n* = 29; 38%).

**TABLE 1:** Summary of clinical areas for SLT practitioners.

Clinical areas^*^	SLT practitioners (*N* = 77)	
	*n*	%
Autism	55	71
Learning disabilities	45	58
Developmental language disorder	34	44
Augmentative & alternative communication	32	41
Dysphagia	30	39
Acquired neurological disorders	28	36
Developmental Speech disorders	27	35
Hearing impairment	22	28
Profound and multiple learning disabilities	21	27
Motor speech disorders	20	26
Dementia	19	25
Stuttering and cluttering	18	23
Bilingualism	18	23
Progressive neurological disorders	18	23
Voice & vocal tract disorders	14	18
Visual impairment	13	17
Sign language (Deaf)	10	13
Cleft palate & craniofacial disorders	8	10
Congenital deaf‐blindness	6	7
Other	6	7

Clinical areas*	SLT practitioners (*N* = 77)	
	*n*	%
Autism	55	71
Learning disabilities	45	58
Developmental language disorder	34	44
Augmentative & alternative communication	32	41
Dysphagia	30	39
Acquired neurological disorders	28	36
Developmental Speech disorders	27	35
Hearing impairment	22	28
Profound and multiple learning disabilities	21	27
Motor speech disorders	20	26
Dementia	19	25
Stuttering and cluttering	18	23
Bilingualism	18	23
Progressive neurological disorders	18	23
Voice & vocal tract disorders	14	18
Visual impairment	13	17
Sign language (Deaf)	10	13
Cleft palate & craniofacial disorders	8	10
Congenital deaf‐blindness	6	7
Other	6	7

SLT educators: There were only three respondents (lecturer = 1; lecturer‐researcher = 2;). In terms of their academic/professional disciplines, all 3 respondents cited SLT with 2 citing linguistics/psycholinguistics also. Subject areas for teaching included linguistics/psycholinguistics (*n* = 2); sociolinguistics (*n* = 2); phonetics/phonology (*n* = 2); education (*n* = 1) and SLT (*n* = 3). The respondents had been involved in teaching SLT students 4–15 years. Two respondents focused exclusively on children and one on children and adults. They collectively covered a range of clinical areas in their teaching: developmental language disorders (*n* = 3); bilingualism (*n* = 2; autism (*n* = 1); learning/intellectual disabilities (*n* = 1); deafness and hearing impairment (*n* = 1); literacy, dyslexia and reading difficulties (*n* = 1. ‘Other’ included ‘linguistics and language’.

### 3.2 Models/Frameworks Adopted

SLT Practitioners: Therapists were asked if they used any models of understanding in their work. Just over half responded affirmatively (*n* = 40; 52%). As shown in Table 2, there was a wide range of responses, the most popular being Marion Blank's levels, models of understanding integrated in an intervention (e.g. DLS: Knowles and Masidlover 1982), neuro‐cognitive models and general models of language development.

**TABLE 2:** Summary of models and frameworks referenced by respondents (SLT practitioners: *n* = 42).

Models/frameworks	*n*	Summary
The Blank Language Scheme (also referred to as Blank's language levels) (Blank et al. 1978)	16	Language is conceptualised across four developmental levels: (1) matching experience, (2) selective analysis of experience, (3) reordering experience, and (4) reasoning about experience. These levels form the theoretical foundation of the Pre‐school Language Assessment Instrument, which is designed for use with children aged 3 to 5 years
Derbyshire language scheme/information carrying words (ICWs)/key words (https://www.derbyshire‐language‐scheme.co.uk)	14	A language intervention programme focusing on the child's use and understanding of language, it is based on assessment of how many ICW units a child can process from verbal instructions. It ranges from 0 ICWs (entirely reliant on contextual cues) to over 4 ICWs, with intermediate levels typically between 1 and 3.
Cognitive neuro‐psychology model of language function (Kay et al. 1996)	8	Developed by Kay et al. (1996), the cognitive model of *language function* organises language processing around distinct modules within a whole language system. Each module may be impaired independently, resulting in specific language deficits. A battery of tests comprises the Psycholinguistic Assessments of Language Processing in Aphasia (PALPA: Kay et al. 1992), to measure a range of language abilities.
Elklan's Communication Chain (https://www.elklan.co.uk)	3	This illustrates the processes and some of the key skills involved in understanding and conveying a message between a listener and a speaker. It is used in Elklan Training to support the development of children's speech, language and communication.
Pyramid of language development (see Morgan and Dipper 2018).	3	This is frequently cited as a visual model of typical language development but no single source is evident. It is used to support explanations to parents, caregivers and health and education professionals—without specialist training in speech and language therapy.
Psycholinguistic model (Stackhouse and Wells 1997)	2	A psycholinguistic model is adopted to investigate the underlying nature of children's speech, language and/or literacy difficulties and to devise targeted intervention based on those difficulties.
Hanen (https://www.hanen.org/home)	2	The Hanen Centre offers a portfolio of programmes designed for parents, caregivers, educators and speech and language therapy professionals to promote children's social, language and literacy development during everyday interactions and routines
Talking Mats (https://www.talkingmats.com)	2	A Talking Mat is a visual communication framework using picture symbols and simple rating scales within a defined physical or digital space. It supports individuals with communication difficulties to express their views and preferences on specific topics or propositions.
Cognitive‐communicative competence model (McDonald 2017)	2	The cognitive‐communicative competence model focuses particularly on people with acquired brain injury (ABI). The model provides a holistic view of communicative competence as a ‘multi‐faceted construct’ affected by a complex array of factors. Auditory comprehension is identified in one of the four domains that make up the model.
Gestalt language processing	2	Gestalt language processing originates from the work of Prizant (1982) on the communicative development of autistic children and adults. It refers to the use of imitative phrases—often echolalic, in which children repeat chunks of language they have heard. Over time, these stored phrases are broken down and recombined, allowing the child to generate novel utterances based on familiar language patterns.
Models of self‐awareness (Pyramid model of self‐awareness: Crosson et al. 1989; dynamic comprehensive model of self‐awareness, Toglia and Kirk 2000; Toglia and Goverover 2022)	2	A multi‐dimensional model of self‐awareness comprising hierarchical levels of: intellectual awareness; emergent awareness; and anticipatory awareness (Crosson et al. 1989). This hierarchical structure was later revised by Toglia and colleagues (2000; 2022), who reconceptualised it as a dynamically interconnected model. Both frameworks aim to capture the extent to which an individual is aware of their own difficulties and how these challenges affect their daily functioning.

Single references were made to a range of approaches and models. Intervention approaches were identified including: the social interaction model used by the Talkabout Social Communication Skills package (Kelly 2016); Positive Behaviour Support (originating from Applied Behaviour Analysis: Carr et al. 2002)—a person‐centred approach offered to people with learning disabilities and/or autism to support behaviour change; Intensive Interaction, which promotes social inclusion and communication with people with profound and multiple learning disabilities (PMLDs) (https://www.intensiveinteraction.org); the curriculum‐based approach *Routes for Learning* for the assessment and support of learners with PMLDs (https://hwb.gov.wales/curriculum‐for‐wales/routes‐for‐learning). One respondent referenced the UK Accessible Information Standard which is encased in law (July 2016: Section 250 of the Health and Social Care Act, 2012) and focuses on meeting the communication and information support needs of individuals with a disability or sensory loss (https://phescreening.blog.gov.uk/2016/08/15/accessible‐information‐standard‐ensuring‐equal‐access‐to‐screening). There were also single mentions of individual concepts or principles: Gricean maxims or the rule people follow in conversation (Grice, 1975); arousal curve (e.g. Burbridge et al. 2005; Kuperman et al. 2014); total/inclusive communication (Jones 2000; Luckins et al. 2025); and executive functioning (Shokrkon and Nicoladis 2022); Bucket Theory of interactions between linguistic domains (Crystal 1987).

SLT educators: All three respondents viewed understanding as a component within the architecture of language and communication and not as a separate entity. Models of understanding cited, included Blank's levels and the DLS/information carrying words (Knowles and Masidlover 1982) (*n* = 2). Models of inferential comprehension were particularly cited by one respondent, for example, Dawes et al. (2018); Filiatrault‐Veilleux et al. (2016); Crystal's (1976) Language Assessment: Remediation and Screening Procedure (LARSP) was identified in relation to teaching about syntactic understanding.

### 3.3 Assessment of Understanding

SLT practitioners: Table 3 summarises the different aspects of speech, language and communication that are assessed by SLTs in their practice. The results are presented in rank order according to the sum of their weighted scores.

**TABLE 3:** Aspects of speech, language and communication that are assessed in rank order: *n* (weighted).

Aspects targeted for assessment	Usually (3)	Often (2)	Sometimes (1)	SUM of weighted scores	Never
Attention levels	47 (141)	21 (42)	8	**191**	1
Vocabulary, lexicon & semantics	40 (120)	20 (40)	14	**174**	3
Information carrying words	37 (111)	21 (42)	15	**168**	4
Response to different communication modalities	33 (99)	25 (50)	16	**165**	3
Language processing aspects (e.g. grammar & complex language)	29 (87)	27 (54)	17	**158**	4
Response to situational cues	29 (87)	26 (52)	19	**158**	3
Pragmatics & social understanding (e.g. conversation skills, rules, topic management, inference)	37 (111)	23 (46)	14	**157**	3
Interactive partners’ communication styles, levels, adaptations	26 (78)	26 (52)	19	**149**	6
Response to abstract language	25 (75)	27 (54)	19	**148**	6
Connected discourse (narrative, exposition)	16 (48)	18 (36)	29	**113**	14
Socio‐cultural aspects	11 (33)	22 (44)	35	**112**	9
Response to sensory cues (e.g. visual, auditory, haptic, olfactory, movement)	10 (30)	24 (48)	24	**102**	19
Reading comprehension	12 (36)	15 (30)	27	**93**	23
Response to prosody	4 (12)	14 (28)	35	**40**	24
Response to aesthetic aspects of language, e.g. poetry	0	2 (4)	17	**21**	58

The aspect ‘attention levels’ was the most frequently targeted aspect for assessment (sum = 191), followed by ‘vocabulary and semantics’ (sum = 174). Next in rank order were ‘information carrying words’ (sum = 168) and ‘communicative modalities’ (sum = 165). ‘Language processing’ (sum = 158), ‘response to situational cues’ (sum = 158) and ‘pragmatics and social understanding’ (sum = 157) were below these. The two aspects achieving the lowest ranked sum weighted scores were: ‘response to prosody’ (sum = 40) and ‘response to aesthetic aspects of language’ (sum = 21).

SLT educators: All aspects of speech, language and communication were addressed in their teaching about understanding to lesser or greater extents. The exceptions were ‘response to aesthetic aspects of language’, which none of the participants addressed, and ‘response to prosody’ and ‘response to sensory cues’ which one participant said they did not include in their teaching.

### 3.4 Assessment Tools

SLT practitioners: As shown in Table 4, recommended *assessment* tools and procedures used to establish an individual's competence for understanding identified three major approaches: bespoke tools, published tools and informal approaches. Most respondents used more than one type of approach or tool. One respondent reported not using any tools due to the particular nature of their work.

**TABLE 4:** Recommended assessment tools/approaches for investigating understanding (*N* = 77).

Bespoke tool				Published tool						Informal							
Service or individual		Repurposed approach		Standardised		Criterion‐referenced		Programme checklist		Patient‐reported outcomes		Interview		Observation		Conversation	
***n***	**%**	***n***	**%**	***n***	**%**	***n***	**%**	***n***	**%**	***n***	**%**	***n***	**%**	***n***	**%**	***n***	**%**
28	36	7	9	65	84.4	55	71.4	23	29.9	1	1.2	2	2.6	10	12.9	2	2.6

Published tools were identified most frequently, and included assessments that are both standardised (84.4%) and criterion‐referenced(71.4%). Programme checklists were used by 29.9% of the respondents and only one reported using a patient self‐reported outcomes tool. Two types of bespoke tool were used: a service or individual‐based tool developed in a specific locality and published approaches that had been repurposed for a particular situation. Informal assessment approaches included interview with relevant professionals, family members and carers, and the individual themselves, observation and open conversation.

SLT educators: Various published assessment tools were identified by each of the respondents, which included batteries such as the Clinical Evaluation of Language Fundamentals (CELF: Semel et al. 2003), Psycholinguistic assessments of language processing in aphasia (PALPA: Kay, Lesser and Coltheart 1996), the Test for Abstract Language Comprehension (TALC: Elks and McLachlan 2012), and the British Picture Vocabulary Scale (BPVS: Dunn 2011). Informal methods such as interview of significant others (e.g. other professionals including teachers and nursing staff, family members and carers), analysis of naturally occurring conversations and observations were also highlighted.

### 3.5 Discrepant Views on Understanding

SLT practitioners: Respondents were asked if they had ever encountered a situation where families, carers and staff had a view about a person's understanding that differed from that of the therapist. This proved to be common, with 49% reporting such differences as frequent, and 43% as happening sometimes (92% overall).

The possible reasons for differing views about a person's understanding were attributed to five main themes as shown in Table 5: the *environment* in which communication takes place; the *setting culture*; characteristics of *significant others* related to the person with understanding difficulties (e.g. other professionals including teachers and nursing staff, family members and carers); *person‐centred* focused on individual characteristics; and *assessment* approaches used to determine levels of understanding.

**TABLE 5:** Summary of suggested reasons for discrepant views on a person's competence for understanding (*N* = 77).

Environment		Setting culture		Significant others		Person‐centred		Assessment	
***n***	**%**	***n***	**%**	***n***	**%**	***N***	**%**	***n***	**%**
32	41.5	3	4	77	100	30	39	27	35

The *environment* was defined by the routines and activities taking place and other contextual factors such as ambient noise level and visual distractions. In particular, routines and situational cues were identified as critical to understanding, but also associated with possible masking of specific problems with linguistic understanding.

‘Not recognising the cues in the environment such as routine, gestures, objects.’ [Participant 5]

The contrast between a controlled setting for communication and the typical classroom and its effect on understanding was highlighted.

‘…the environment (quiet 1:1 clinic room versus loud, busy, noisy classroom)…’ [Participant 59]

*Setting culture* was referenced by only three participants. It was defined in terms of a closed culture that did not admit other professionals or ways of looking at a person's understanding.

‘…Hostility of environment’ [Participant 01]

which was also attributed by one respondent to:

‘…lack of respect for SLT role and knowledge and skills’ [Participant 24]

The role of *significant others* (e.g. other professionals including teachers and nursing staff, family members and carers) and their different perceptions of a person's understanding was cited most frequently as the underlying reason for disagreement about a person's understanding. Over‐ and under‐estimation of a person's capacity for understanding featured, sometimes based on their familiarity with the person or the person's use of social skills.

‘…doctors and nurses often take a patient nodding and giving social greetings as a sign of understanding’ [Participant 28]

The pain of an acquired language disorder and what it means for both the individual and those around them were identified factors associated with the significant other's over‐estimation of their relative's understanding ability.

‘Loss of understanding/comprehension level can feel too painful for relatives to acknowledge as it's suggestive to them of the person losing some of their intelligence and/or their original personality?’ [Participant 13]

The psychological state of significant others in relation to fatigue and work ‘burnout’ was cited by two respondents as a possible reason for paid staff attributing a breakdown in understanding to service user choice.

‘Staff burnout leading to compassion fatigue and feelings that a service user is choosing not to understand/listen.’ [Participant 02]

Sometimes a lack of opportunity for close one‐to‐one interaction was viewed as a factor in the significant other's over or under‐estimation of an individual's skills in understanding.

‘Families and carers not spending significant time with a patient.’ [Participant 54]

The lack of awareness and knowledge of understanding and its development was a frequently cited cause of differences of opinion between SLT and significant others (e.g. other professionals including teachers and nursing staff, family members and carers).

‘Not fully understanding what understanding ‘looks like’ in an education context’ [Participant 07]

*Person‐centred* characteristics were identified as contributing to different perceptions of a person's understanding. These included other competing behaviours and difficulties that appeared to over‐shadow problems with understanding.

‘Mental Health diagnosis masking underlying communication needs’ [Participant 23]

In addition, it was considered that the individual's use of coping or compensatory strategies might mask an underlying difficulty.

‘…The person masks well in some settings e.g. following routines in care homes’ [Participant 16]

The type of assessment approach was considered to be a factor in producing different outcomes sometimes leading to different perceptions of a person's understanding. Participant responses revealed tensions between: the outcomes of different assessment approaches (e.g. use of a standardised tool compared to a more functional assessment); the different opinions of significant others (e.g. the opinion of the SLT as opposed to other professionals including teachers and nursing staff, family members and carers); the context where the individual's understanding is evaluated (e.g. school or home); communication needs in bilingual individuals. In addition, confusion over mental capacity was mentioned as a factor affecting how successfully a person's understanding was determined.

SLT educators: All three respondents recognised the phenomenon of discrepant perspectives on a person's understanding and this was variously attributed to environmental cues and social routines masking true understanding. It was also acknowledged that individuals may respond differently in a formal testing situation compared to everyday social communication.

### 3.6 Aspects of Understanding

SLT practitioners: As summarised in Table 6, working definitions and approaches to explaining understanding referred most frequently to different aspects of the architecture of communication: modalities (*n* = 39; 50.6%); language—grammar, vocabulary and semantics (*n* = 34; 44.2%); functions (*n* = 32; 41.5); inference (*n* = 31; 40.2%). Identification of social interaction and participation was much less frequent (*n* = 7; 9%) with only one participant identifying prosody.

**TABLE 6:** Summary of how understanding is defined and explained to other professionals, family members and carers (*N* = 77).

Themes and sub‐themes	*n*	%
Communication		
Modalities	39	50.6
Language (vocabulary, grammar, semantics)	34	44.2
Functions	32	41.5
Prosody	1	1.3
Social interaction & participation	7	9
Inference	31	40.2

Environment		
Context	13	16.8
Partner skills	1	1.3

Explanatory approach		
Model or framework	35	45.4
Co‐construction	5	6.4
Visual support	5	6.4

Associated factors		
Emotional state, cognition, attention	4	5.1

Asked how SLTs negotiated with significant others (e.g. other professionals including teachers and nursing staff, family members and carers) when discrepant views on understanding were expressed, the respondents described using frameworks that referred variously to development, language processing and the relationship between reception and expression (*n* = 35; 45.4%). Approaches to explanations given to fellow professionals, carers and family members included visual aids (e.g. diagrams) (*n* = 5; 6.4%) and negotiated explanation with a significant other (*n* = 5; 6.4%). Associated factors (emotional state, cognition and attention) and their interaction with understanding were also considered (*n* = 4; 5.1%).

In response to the question about explaining to a fellow professional or family member or carer, both the ability to follow words alone and the ability to follow words through use of contextual cues were highlighted.

‘Not only do we need to understand language, but we also need to understand the behaviours and actions of other people in social situations.’ [Participant 45]

It was recognised that the results of a standardised or formal assessment may not necessarily equate with the views of others, for example, parents, teachers and support staff.

‘No reason to think that professionals know more about understanding than family members.’ [Participant 12]

Working through practical examples was suggested by some respondents as a way of demonstrating how context may affect a person's understanding.

‘…examples of where the person made errors in an assessment, but would also try to relate that back to real life …’ [Participant 15]

SLT educators: The respondents acknowledged the importance of listening to and respecting the different perspectives on a person's understanding, and explaining how situational understanding operates to support language understanding.

## 4 Discussion

### 4.1 Summary

Amongst the 77 SLT practitioner respondents there was a good representation of different clinical areas/specialisms and client populations. Understanding was defined using a range of communication‐related concepts, including modalities; language (vocabulary, grammar and semantics); functions; and the role of inference. The ‘environment’ defined by ‘context’ and ‘partner skills’ was considered less important to the way ‘understanding’ was defined and explained. Approaches to assessment focused on key aspects of understanding such as vocabulary and semantics, and responses to different communicative modalities, language processing and semantics. Attention was most frequently cited within the range of assessment areas. The majority of the respondents used published assessment tools, both standardised and criterion‐referenced, but locally produced, bespoke tools were also deployed. Differing views on a person's understanding were recognised to be a common issue by most SLTs, and were largely attributed to factors associated with other professionals, family members and carers. Other factors mentioned included the environment and the role of situational cues, the degree of person‐centredness of the assessment, and the type of assessment. Where assessment of an individual's understanding by another person (e.g. parent, partner, relative, carer, teacher) was incompatible with that of the SLT, models or frameworks were invoked as part of the explanatory approach adopted. The very small sample of SLT educators, whilst in no way considered representative of the target population, gave responses that were consistent with those by the SLT practitioners.

Various issues that are critical to SLT practice arise from this survey: conceptualisation of understanding; approaches to assessment and intervention, and differing perspectives.

### 4.2 Conceptualisation

Both intrinsic and extrinsic factors were identified as critical components to comprehension, which is consistent with Wolvin's view that neither operate in isolation (2009). The environment (e.g. context, partner skills) had fewer mentions compared to components within the speech, language and communication architecture, although the role of inference was highlighted, which implicates the partner as well as the speaker (Sperber and Wilson 2012). Responses to the question about underpinning models and frameworks for understanding varied, but the most striking finding was that nearly half of the respondents cited no models at all. Those models that were identified were typically aligned to different client groups in focus, for example, Blank Language Scheme/Blank's levels (Blank et al. 1978) and the DLS (Knowles and Masidlover 1982) to the developmental field; the PALPA (Kay et al. 1996) to the adult acquired field. Reference was made to other models used in practice but their specific relevance to comprehension was not clear, for example, the pyramid model of self‐awareness (Crosson et al. 1989); dynamic comprehensive model of self‐awareness (Toglia and Kirk 2000; Toglia and Goverover 2022). Usability may be a factor in the selection and use of particular models. For example, Blank's simple framework provides an immediate way of looking at and categorising the individual's responses in context. Thus the deliberate linking of theory and practice in a publication may popularise its use amongst practitioners.

Other answers referenced unitary concepts such as ‘arousal’ or ‘executive function’, and intervention approaches that entailed participant understanding (e.g. Talking Mats: Murphy and Cameron 2008). It is possible that the words ‘model’ or ‘framework’ have ceased to have currency in clinical practice as practitioners focus on the perceived needs of individual clients. This explanation resonates with Goldbart et al.’s (2014) report of the inconsistent practice of SLTs with persons who have profound intellectual and multiple disabilities where research evidence was also largely neglected, and with Morgan and Dipper's (2018) finding that intuition and experience appear to count for more than theoretical frameworks. This dissociation is reflected in the broader context of clinical practice where a research‐practice gap remains between theory, evidence gathering, and everyday interventions (Rapport et al. 2022). Alternatively, there may be some confusion around what is understood by ‘model’, ‘theory’ and ’framework’. Rapport et al. (2022) refers to: ‘model’ as a guide for the use of evidence in practice; ‘theory’ to explain the ‘causal mechanism’ for the condition or the change process in therapy; and ‘framework’ for identifying factors of influence over outcomes.

Another term which benefits from increased specification is ‘context’. Ferstl (2021) in a review of the literature, identifies four distinct categories: co‐text; linguistic context; cognitive context (mental model); social context. Ferstl describes the application of different contexts in psycholinguistics and pragmatics, but concludes that as yet such integrated approaches are at an early stage.

The advancement of holistic therapy in the mid‐2000s (e.g. McLeod 2006; Stemple 2005), whereby formal analytical practices are combined with informal approaches such as environmental observation and identification, may be relevant here. The holistic view defines verbal comprehension as multi‐faceted and associated with various inter‐linked competencies, where intrinsic and extrinsic factors are enmeshed. Thus, to cite an analytical framework or model might be considered to overlook critical extrinsic factors. The person is located at the centre of SLT practice, which might explain the references made to broader psychosocial models and frameworks, and a variety of approaches that emphasise the complex, dynamic, interactive nature of language comprehension (e.g. Azios et al. 2023; Grove 2014; Horton et al. 2020; Prizant et al. 2017; Wilkinson 1999).

### 4.3 Assessment

The data captured the range and diversity of approaches taken to assess and address understanding difficulties. Assessment emphasised ‘attention levels’ and individual components of the language architecture (e.g. ‘vocabulary and semantics’) as well as ‘response to situational cues’ and ‘pragmatics and social understanding’. Thus it would appear that many therapists think of understanding in the broader context of psychological processes underpinning adaptive behaviour (e.g. Krauzlis et al. 2023), whilst conscious that ‘attention’ has also been identified as a ‘conceptually fragmented’ term, freighted with many meanings (Anderson 2023). This is consistent with the findings of Wallace et al. (2022) who conducted a meta‐review of papers and identified no less than 28 interventions, categorised as those that isolated auditory verbal comprehension as a target, focusing on lexical and semantic aspects and requiring only nonverbal responses (29%); mixed approaches that targeted auditory input but added a different modality, for example, writing, gesture (46%) and indirect treatments, focusing on underlying skills such as attention or working memory (25%).

In addition to a range of clinical assessment tools, bespoke tools, either service based or individually generated, were specified by approximately a third of the respondents, and included interviews and checklists. Although informal assessments may provide an important adjunct to formal assessment procedures, without having a clear idea about how these types of assessment are structured, how data are recorded and analysed, it is difficult to assess how much they contribute to our knowledge of a person's understanding. Part of the motivation to use informal assessment methods may be to capture a more functional view of the client's communication skills. For example, conversation analysis has been used to identify ‘trouble’ in conversations and approaches to conversational repair across various client groups including: people with aphasia (Tetnowski et al. 2021); people with acquired brain injury in hospital (Foulkes et al. 2024); older people with dementia (Chatwin et al. 2024) and people with intellectual disabilities (Jingree et al. 2006). However, conversation analysis was rarely mentioned by respondents. We suspect this may be due to the time required to capture conversations, produce a detailed dialogic transcription and engage in the required inductive analysis.

The focus for assessment of a person's understanding was predominantly aligned to intrinsic aspects of the person's language and communication architecture (e.g. attention levels, lexicon, morpho‐syntax and pragmatics) rather than extrinsic factors (e.g. conversation partner style, socio‐cultural aspects). The dominant ethos seems to be a deficit model—that is, the emphasis is placed on what a person fails to understand, rather than the adaptive strategies they use in order to function in everyday life. Thus it seems that practitioners focus on structural aspects of language and communication in their assessments, and sideline psychosocial factors, although they may refer to these in holistic profiling and in defining appropriate interventions.

The two aspects achieving the lowest ranked sum weighted scores were: ‘response to prosody’ and ‘response to aesthetic aspects of language’. It was expected that aesthetics would be an unlikely priority for SLTs. However, it is increasingly the case that individuals regarded as having clinical language impairments can achieve success in creative performance and creative writing, so that observations of response to pattern and beauty in language are not perhaps as irrelevant as might appear (Bunning and Grove 2024).

The lack of attention to prosody is somewhat concerning. Goswami (2022), argues that speech rhythm patterns are fundamental in building the ‘linguistic brain’ Prosodic cues help infants to segment speech (Nazzi et al. 2000); are interpreted as cues for turn‐taking and signal pragmatic meanings (Esteve‐Gibert and Prieto 2018; Giberga et al. 2025; Kalashnikova and Kember 2020). Prosody is a critical route to inferences about the speaker's emotional state (Aguert et al. 2013). The prosody of infant directed speech patterns seems associated with positive outcomes for both attention and communication (Spinelli et al. 2017). In clinical populations the ability to discern and apply prosodic information has been shown to be problematic for children with autism (Grice et al. 2023) and those with developmental language disorders (Fisher et al. 2007; Parvez et al. 2024). In aphasia, both understanding and production of prosody may be affected (Jiang et al. 2024; Zipse et al. 2025). Prosodic cues are also vital in rehabilitation and intervention (Giberga et al. 2025; LaCroix et al. 2020).

### 4.4 Discrepant Views

The current research establishes that discrepant views of a client's understanding are indeed a major clinical issue, encountered by 92% of SLTs. Where SLT assessment of a person's understanding ran counter to the views of others (e.g. other professionals including teaching and nursing staff, family members and carers), all the respondents cited factors associated with the significant others. Incidents of conflicting assessments of a person's comprehension between SLT and significant other clearly happen. This would appear to challenge Purse and Gardner's (2013) report of convergent assessment outcomes for SLTs and teachers. Occurrence of significant others (e.g. other professionals including teachers and nursing staff, family members and carers) over‐estimating and under‐estimating a person's comprehension were described by the respondents, who provided a variety of reasons for the divergence of opinions. These included: use of situational cues to mask difficulties (cf, Vasiljevic et al. 2025); staff acceptance of non‐verbal responses (e.g. nodding) as evidence of verbal comprehension; and the need for significant others (e.g. other professionals including teaching and nursing staff, family members and carers) to take a positive view of the person's abilities. Lack of knowledge of language processes, and the opportunity to conduct in‐depth assessment of comprehension were identified as root causes of such divergent opinions. Also mentioned by respondents and identified by Vasiljevic and colleagues (2025) is ‘masking’ or hiding of difficulties. This can be a factor when people with aphasia are reluctant to disclose their difficulties to new people they encounter in social situations, for fear of stigma.

Just under 50% of respondents stated that they used models and frameworks to explain the results of the SLT assessment despite the aforementioned limited reference to models in their work. Conversely, the environment defined by context and partner skills was rarely cited. This resonates with the findings of Morgan et al. (2019) who reported that therapists see work with families and carers as vital but do not necessarily specify how they work with them.

### 4.5 Strengths and Limitations

This is the first study to look at the conceptualisation of understanding in SLT practice. To address the first research question, two surveys were planned to run in parallel: one for SLT practitioners and one for SLT educators. Unfortunately, there were only three respondents to the second survey. This is disappointing, since little is known of what students are currently taught about this topic. It is hoped that as a result of this paper, educators may be more inclined to address this question. A revised version of the survey aimed at educators is planned for a later date.

The sample of 77 SLT practitioners comprises only a minority of the profession although they appeared to represent a reasonable cross section, distributed fairly evenly across both experience levels and clinical groupings. However, it may be that the sample is skewed to those who encounter misunderstandings between SLT and significant others and who have an active interest in the problem of conceptualising understanding. Nevertheless, as a first exploratory probe, the survey appears to have captured an authentic spread of clinical areas and experience.

With regard to the questionnaire content, supporting definitions of key terms used in the questionnaire (e.g. model, framework, theory, approach) might have helped respondents to provide more fully articulated answers. This may also be applicable to the question about socio‐cultural aspects. However, at a time when the decolonisation of the profession is active and encouraged by RCSLT (see www.rcslt.org) it was expected that more attention would be paid to this dimension. Without understanding cultural norms of dialogue, it is impossible to effectively assess or work with families and staff. The absence of references to conversation analysis and linked remediation techniques was disappointing; however, it is possible that if analysis of dialogues with carers had been included as a clinical area, there would have been a higher response rate regarding naturalistic assessment and intervention approaches. For example, the use of video interaction guidance has gained in popular usage over the last 10 years (https://www.videointeractionguidance.net/what‐is‐vig).

The questionnaire used a basic MS Forms platform, which produces a summary report of the whole data set but lacks the facility to identify individuals and to correlate variables. For example, it would have been useful to explore respondent post‐qualification years of experience in relation to models and frameworks adopted. For future surveys, it is recommended that a more powerful platform such as ‘qualtrics’ (https://www.qualtrics.com) is used.

### 4.6 Conclusions and Implications

Conceptualisation of understanding and its operationalisation in the ongoing practice of SLTs appear to vary. Without a clearer picture of what is happening in pre‐registration studies, it is difficult to assess the place of theory in SLT practice. The temporal distance from completion of pre‐registration studies may be a factor in respondent access of models and theories: the majority of the respondents had four or more years’ experience of practice, with only 10% of respondents having worked 1–3 years post‐qualification. Concepts and practices may vary according to client group and practitioner variables. Published assessment tools and intervention approaches are typically designed with a particular client group in mind, and this together with the culture of the setting (e.g. school, hospital or adult community services) may influence the frame of reference for SLT practice.

It is clear that discrepant perspectives do occur between SLT practitioners and others (e.g. professionals, family members and carers) around a person's understanding. Such differences are often attributed to the absence of situational cues in the formal assessment condition and the presence of situational cues in the natural environment. Various reasons for differences in interpretation between professionals and others (e.g. other professionals, family members and carers) have been proposed, which include references to the social context (e.g. culture, relationship to individual); deficit versus strength based attitudes; over‐interpretation by significant others, and masking by the individuals themselves. In conclusion, 6 years on from Morgan et al. 2019) study and 10 years on from that of Goldbart et al. (2014), it seems possible that the SLT profession still faces challenges in locating practice in explicit theory and the available research evidence base. Instead, practitioners maintain a focus on client‐centred presentations and associated factors that are intuitively easier to grasp and apply. This is unsurprising in the light of Ferstl's conclusion (2021) that we are as yet very far from developing a unified theory of understanding that would allow for testable predictions regarding the effects of context on comprehension. There is a clear need to investigate further the theories that underpin SLT practice, and to take seriously Ferstl's suggestion that ‘social cognitive processes’ are a ‘crucial prerequisite’ (p. 69) to theory advancement, based on collaboration between different disciplines, research areas and theoretical perspectives.

Implementation science may have a role in resolving the theory‐practice relationship in speech and language therapy. Evidence‐based practice in health leans towards the gold standard of randomised control trials (Cartwright, 2007). However, even the most comprehensive evidence requires translation into ‘real‐world effectiveness’ (Rapport et al. 2022; p. 992). In separate explorations of decision‐making in speech and language therapy practice, both Greenwell and Walsh (2021) and McCurtin and Clifford (2015) assert the relevance of the real world by placing research evidence with other types of evidence: client‐centred practice or experience focused, and context. Douglas and Burshnic (2019) explain that intervention research is typically conducted in optimal conditions established through use of pre‐planned methods; however, implementation in the ‘real world’ may require modifications. They identify three points of navigation that constitute implementation science: define implementation science as: (a) process description, (b) understanding and explanation of influences on outcomes, (c) evaluation in real world contexts. These are grounded within a Theoretical Domains Framework, used in this context to conceptualise behaviour change. Understanding of the problem of translating evidence to practice is therefore fundamental in implementation science: we suggest that we also need to pay attention to the way clinicians interpret and address the nature of a communication problem like understanding, such that theory is mobilised and practice improved (Rapport et al. 2022). The extent to which SLT curricula embrace research evidence alongside systematic implementation is a question that stems from Rapport et al. (2022) and one that is ripe for investigation in both academic and clinical practice in the future.

## Ethics Statement

The study received ethical approval from the Faculty of Medicine and Health Ethics Monitor at the University of East Anglia (ETH2324‐1590).

## Conflicts of Interest

The authors declare no conflicts of interest.

## Data Availability

The data that support the findings of this study are available on request from the corresponding author. The data are not publicly available due to privacy or ethical restrictions.
